# Nutritional management and geno-phenotyping of clinical nutrition in patients with glycogen storage diseases type VI and IX

**DOI:** 10.1038/s41430-025-01614-0

**Published:** 2025-04-10

**Authors:** Sema Kalkan Uçar, Alperen Elek, Havva Yazıcı, Yasemin Atik Altınok, Ayşe Yüksel Yanbolu, Fehime Erdem, Merve Yoldaş Çelik, Ayça Aykut, Asude Durmaz, Ebru Canda, Mahmut Çoker

**Affiliations:** 1 Department of Pediatrics, Division of Metabolism and Nutrition, Ege University Medical Faculty, Izmir, Turkey; 2 Ege University Medical Faculty, Izmir, Turkey; 3 Department of Genetics, Ege University Medical Faculty, Izmir, Turkey

**Keywords:** Dyslipidaemias, Phase IV trials

## Abstract

**Background/Objectives:**

Glycogen storage diseases type VI (GSD-VI) and type IX (GSD-IX) are rare inherited metabolic disorders caused by enzyme deficiencies that disrupt glycogen metabolism. The aim of this study was to analyze the clinical features, nutritional management and geno-phenotyping of clinical nutrition in a cohort of patients with GSD-VI and GSD-IX.

**Subjects/Methods:**

A retrospective cohort study was conducted with 16 patients with GSD-VI and GSD-IX. Demographic characteristics, clinical and laboratory findings, and nutritional treatment outcomes were collected and analyzed.

**Results:**

The mean patient age was 10.57 years (±4.81). The distribution of the diagnoses was as follows: GSD-IXa (3), GSD-IXb (6), GSD-IXc (1), and GSD-VI (6). The average age at diagnosis was 36.5 months (±42.2) (13–114 months) in the GSD-VI group. Among the GSD-IX subgroups, the mean age at diagnosis varied: 23.3months (±4.16) for GSD-IXa, 35.7months (±17.5) for GSD-IXb, and 78months for GSD-IXc. Over the course of the study (4.5 ± 1.77 years), protein intake in GSD VI patients increased by 1.05 g/kg/day (91.3% increase), while in GSD IX patients, it rose by 1.09 g/kg/day (94% rise). Uncooked cornstarch (UCS) started at 1 g/kg/day for GSD-VI and 0.85 g/kg/day for GSD-IX, later reduced to 0.71 g/kg/day (29% decrease) and 0.52 g/kg/day (60% reduction), respectively.

**Conclusion:**

Overall, this paper provides valuable insights into managing GSDVI and GSDIX patients, emphasizing the role of a high-protein diet aligned with the disease’s pathophysiology and the potential of genotyping to enhance nutritional treatment protocols.

## Introduction

Glycogen storage diseases (GSDs) are a group of inherited metabolic disorders that affect the body’s ability to mainly use and rarely to store glycogen properly. There are several types of GSDs, each caused by a specific enzyme deficiency that disrupts glycogen metabolism [1]. While GSD-VI (also known as Hers disease, MIM #232700) is rarely encountered, GSD-IX (IXa, MIM #30600; IXb, MIM #261750; IXc, MIM #613027) is one of the most common types of GSDs [2].

GSD-VI is caused by mutations in the *PYGL* gene, located in the 14q21-q22.3 region of chromosome 14. This disorder is inherited in an autosomal recessive pattern, and the prevalence of GSD-VI is approximately 1 in 100,000 individuals [3]. The *PYGL* gene provides instructions for making the liver phosphorylase enzyme [4]. GSD-IX is caused by a deficiency in the multi-subunit enzyme complex called phosphorylase kinase (PhK). The complex is composed of subunits encoded by specific genes, including *PHKA1, PHKA2* for the α subunit, *PHKB* for the β subunit, *PHKG1, PHKG2* for the γ subunit, and *CALM1, CALM2, CALM3* for the δ subunit. Based on it, among GSD-IX subtypes, GSD-IXa is liver-specific, caused by *PHKA2* mutations in the α subunit with X-linked inheritance (MIM #306000). It has two forms: one with PhK deficiency in both liver and red blood cells (RBCs), and another where liver PhK activity varies, remaining normal or elevated in RBCs. GSD-IXb, linked to *PHKB* mutations in the β subunit, and GSD-IXc, caused by *PHKG2* mutations in the γ subunit, are both autosomal recessive (MIM #261750 and #613027, respectively) [3].

Both, GSD-VI and GSD-IX typically manifest in early childhood, with most patients exhibiting hepatomegaly. Elevated liver enzymes are also often present. As the disease progresses, patients may develop severe hypoglycemia, short stature, mild gross motor delays, liver cirrhosis, and liver disease. In addition to these symptoms, rare findings such as cardiomyopathy, hepatocellular carcinoma, muscular hypotonia, and post-prandial lactic acid elevation have been reported in the literature [1, 2, 5–9].

Differentiating GSD-IX from GSD-VI is challenging due to their similar clinical presentations [3]. Next-Generation

**Fig. 1:**
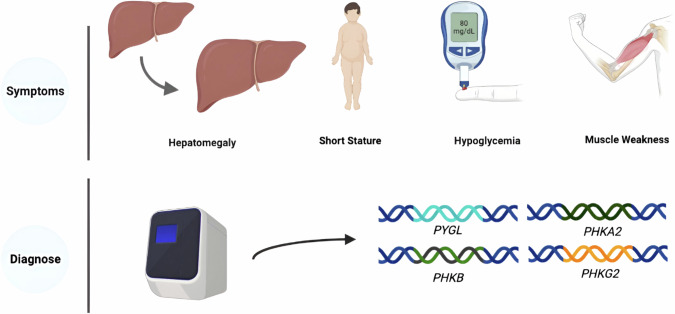
This illustration, provides an overview of the clinical presentations and diagnosis of GSD-VI and GSD-IX. It offers a visual guide to understand the symptomatic manifestation and diagnostic approaches associated with these metabolic disorders. Designed with BioRender.

Sequencing (NGS) techniques are employed to diagnose these diseases by analyzing the genetic mutations that cause them (Fig. 1).

Here, we aim to analyze the follow-up data of GSD-VI and GSD-IX patients expanding phenotypic and genotypic spectrum, disease progression, and particularly nutritional treatment outcomes between GSD-VI and GSD-IX.

## Material and methods

The study was carried out following the Declaration of Helsinki. The patients or their relatives obtained an informed consent form. The study was approved *(approval number:* 23–7.1T/10*)* by the Ethics Committee of Ege University Faculty of Medicine. The study included 16 patients diagnosed with GSD-VI (*n* = 6) and GSD-IX (*n* = 10) at Ege University Faculty of Medicine. Patients were selected based on their medical records and genetic confirmed diagnoses.

Demographic characteristics, clinical presentations, and laboratory findings were collected for all patients. These included age at diagnosis, gender, symptoms at presentation, and liver function tests. Dietary recalls were conducted to assess actual nutritional intake. We collected three days of dietary records—two during the week and one on the weekend—to ensure a comprehensive evaluation of the patients’ usual eating patterns. The nutrient distribution from the dietary intakes was evaluated at both the first and last visits to track any changes over time. Disease progression was monitored over a median follow-up period of 95 ± 41.62 months.

Data were entered into an Microsoft Excel database, which was used for data management and analysis. Differences in clinical features and laboratory findings between the two groups were analyzed using descriptive statistics. The chi-square test or Fisher’s exact test was used for categorical variables, and the Mann–Whitney U test was used for continuous variables. All data were analyzed using SPSS software version 25 (IBM Corp., Armonk, NY, USA), and a *p*-value of <0.05 was considered statistically significant.

## Results

Sixteen patients, including 12 males and 4 females (2 of whom had GSD-IX and 2 had GSD-VI), from 15 unrelated families were retrospectively analyzed. Among the patients, the diagnostic distribution was as follows: 3 (19%) GSD-IXa, 6 (37%) GSD-IXb, 1 (6%) GSD-IXc, and 6 (37%) GSD-VI. The average age at diagnosis for GSD-VI was 36.5 ± 42.2 months (13–114 months). For GSD-IXa, it was 23.3 ± 4.16 months (18–27 months). GSD-IXb had an average age at diagnosis of 35.7 ± 17.5 months (12–60 months). GSD-IXc was diagnosed at 78 months.

### Presentation & outcome of GSD-VI and GSD-IX

#### Clinical features

While hepatomegaly was observed in all patients with GSD-VI, hypoglycemia was observed only in Patient 1. On the other hand, two patients with GSD-IX exhibited both hepatomegaly and hypoglycemia, while Patient 10 exhibited only hypoglycemia (Table 1).

**Table 1:** Clinical, biochemical and genetic data of patients with GSD type VI and IX.

Patient No/Gender/ Gene	Genetic variants	Age at diagnosis/Follow-up period (years)	Initial Symptoms	Weight;Height-SDS (diagnosis/last visit)	Liver Bx	Liver Ultrasound (diagnosis/last visit)	ALT at diagnosis/at last visit (NR: ( < 45IU/L)	TG at diagnosis/at last visit (NR: <150 mg/dl)	Biotinidase at diagnosis/at last visit (NR:4.4–12 nmol/dk/mL)	Treatment UCS (g/kg/d) (at diagnosis-last visit) Protein(g /kg/day %TC) MCT(+/-)
**GSD type VI**										
P1/M/ *PYGL*	c.1131C>G (p.Asn377Lys)	13 mo / 9 y	HM, H	−1.36/−1.1; −1.87/−0.01	S - GSD	HM/HM	80/74	320/131	8.3/5.7	1–1; 1.5–2.5; (+)
P2/M/ *PYGL*	c.1131C>G (p.Asn377Lys)	18mo/14 y	HM	−1.6 / 0.05; −2.2 / −2.0	S – GSD, F	HM, IE/HM	273/216	230/167	8.9/7.4	2–2; 1.2–2.5; (+)
P3/F/ *PYGL*	**c.1806G>C (p.Arg602Ser)**	9.5 y/ 18 y	HM	−1.6/−1.8; −2.2/−1.8	S - GSD	HM, SM/HM	55/10	68/86	7.9/5.6	1–0.6; 0.95−2.0; (−)
P4/M/ *PYGL*	c.1131C>G (p.Asn377Lys)	26 mo/ 11.6 y	HM	−0.03/0.12 0.04/0.4	S – GSD, F	HM/HM, IE	685/67	222/115	6.8/8.7	1–1; 1.1−2.2; (+)
P5/M/ *PYGL*	c.1131C>G (p.Asn377Lys)	2.5 y/ 3 y	HM	0.58/ 0.40; −1.9/−1.05	No	HM/	39/40	317/175	8.2 / 7.1	1–0.5/ 1.2−2.015/+
P6/F/ *PYGL*	**c.1164G**>**A** (p.Trp388*)	20 mo/ 6.5 y	HM	−0.9/ + 0.31 −2.8/ + 0.02	No	HM/	141/42	851/252	8.4/8.6	1–0.7; 0.95−2.0; (+)
***GSD Type IX***										
P7/M/*PHKA2*	**c.1324G**>**A (p.Val442Ile)**	25mo/10 y	HM,H	−2.27/−1.97 −2.24/−0.95	No	HM/HM, IE	70/39	301/98	8.6/6.7	2-Stop; 1.2–2.2; (+)
P8/M/*PHKA2*	**c.321C**>A (p.Trp1067*)	18mo/6.8 y	HM,H	−1.47/−0.51; −2.47/−0.81	S - GSD	HM/ Normal	79/18	166/62	9.2/7.8	3.5−0.7; 1.2–2.4; (−)
P9/M/ PHKA2	**c.1324G**>**A (p.Val442Ile)**	27mo / 12 y	HM	1/−0.9; −2.5/−2	S - GSD	HM/ HM,IE	97/46	316/122	7.7/7.2	2-Stop; 1.2–2.1; (+)
P10/F/ PHKB	**c.594+4T**>**G**	28mo/11 y	H	0.7/ 1.2; 1.2/ 1.2	S - GSD	HM, IE/Normal	18/171	82/48	7.1/7.5	2.3-2; 1.2–2.3 (−)
P11/M/PHKB	c.1969C>T (p.Gln657*)	38mo / 16.5 y	HM	−0.5/−0.5; −2.61/−0.62	S- GSD	HM, IE/ HM, IE	308/25	89/106	7.8/7.2	2–1.5; 1.0–2.3; (−)
P12/F/PHKB	**c.1564C**>T (p.Gln522*)	46mo / 8.5 y	HM	−0.28/1.42; 0.01/1.27	S- GSD, F	HM/HM, IE	104/71	280/105	7.2/12.2	2–1; 1.2–2.1; (−)
P13/M/PHKB	c.1969C>T (p.Gln657*)	60mo/9.1 y	HM	−0.5/−0.87; −0.93/−0.71	No	HM, IE/Normal	100/33	153/206	8.5/6.9	0.5–0.5; 1.0–2.2; (−)
P14/M/PHKB	**c.305+1G**>**A**	14mo / 5.4 y	HM	1.1/0.74; −1.53/0.32	S GSD	HM, HM	121/45	143/102	8.5/8.7	2-Stop; 1.2–2.4; (−)
P15/M/ PHKB	c.1969C>T (p.Gln657*)	12mo/ 8.3 y	HM,H	1.97/ 1.35; 0.95/1.8	No	Normal/Normal	908/22	149/37	9.1/7.9	05-Stop; 1.2–2.5; (−)
P16/M/ PHKG2	**c.112G**>**A (p.Val38Ile)**	78mo/ 19 y	HM	0.5/152; −2.8/−2.7	S GSD, F	HM, IE/ HM, IE	72/30	230/80	6.9/6.42	No; 1.2–2; (−)

The Genetic variants written as Bold have not been reported before.

*P* patient, *M* male, *F* female, *HM* hepatomegaly, *H* Hypoglycemia, *IE* increased echogenicity, *ALT* alanine transaminase, *AST* aspartate transaminase, *mo* months, *y* years, *TG* triglycerides, *UCS* Uncooked cornstarch, *MCT* Medium-chain triglyceride, *Bx* biopsy, *S GSD* suggestive of GSD, *F* Fibrosis.

Half of our GSD-VI patients had short stature (<3p): Patient 3, 5, 6; two of six patients (Patient 1 and 2) were at the borderline (3p). There was an improvement in growth with nutritional therapy in Patient 1, 4, and 6. These patients showed an increase in height percentiles from the 3rd to the 50th percentile, from the 25th to the 75th percentile, and from below the 3rd to the 50th percentile, respectively. Patients 2, 3, and 5 remained at the same growth percentile, showing no decline in their growth measurements. A high-protein diet was applied for an average duration of 4 ± 1.78 years.

**Fig. 2:**
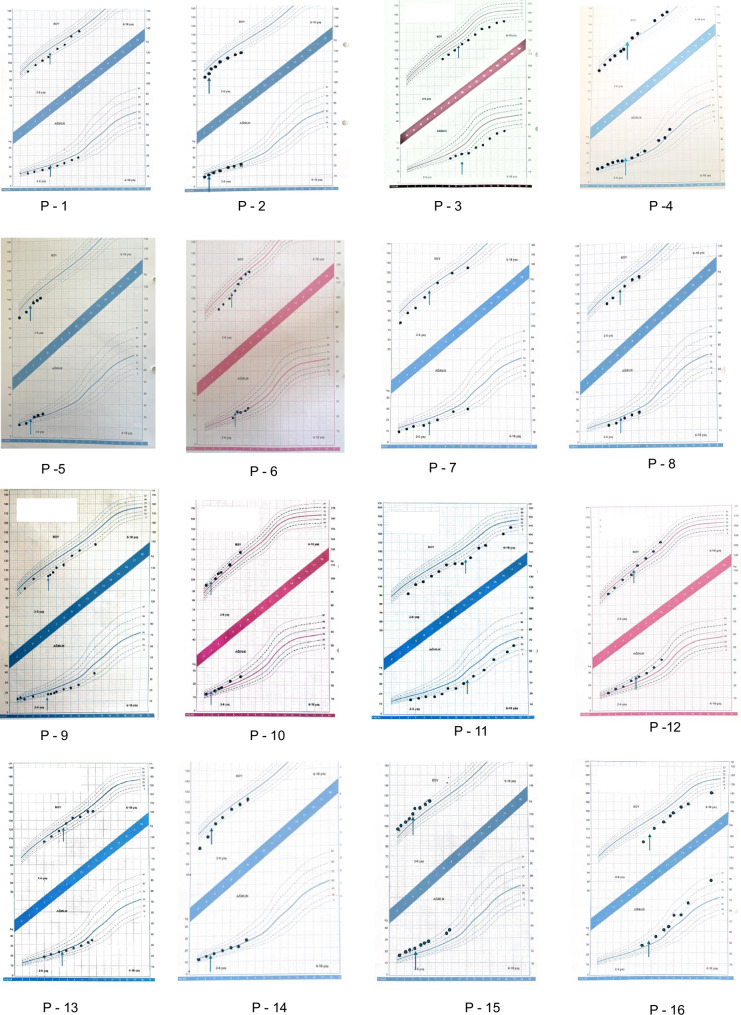
Growth chart of patients diagnosed with GSD- VI (P1-6) and GSD- IX (P7-16). Arrows Indicating the Initiation of High-Protein Diet.

Similarly, half of our GSD-IX patients had short stature (Fig. 2). Patients 7, 10, 11, 12, and 14 showed a significant increase in their height percentiles. Patients 12 and 14 had a prominent increase of the height percentile from 25 to 90 percentile and from <3p to 25 percentile respectively. Patient 8 maintained his growth percentile (10th percentile) throughout the follow-up period. The duration of high-protein dietary therapy for the patients was an average of 4.8 ± 1.79 years.

When comparing the start and end of the follow-up, no decline in growth percentile channels was observed in any patient with either GSD VI or GSD IX, despite some fluctuations during the follow-up period.

#### Consanguinity

Consanguineous marriage was detected in 66% of GSD-VI patients (P1, P4, P5, P6) and in 60% of GSD-IX patients (P9, P11, P12, P13, P14, P16).

#### Liver ultrasound

In initial visit all patients with GSD-VI were found to have an enlarged liver on liver ultrasound. Patient 2 was additionally observed to have increased echogenicity, while Patient 3 was observed to have splenomegaly (as shown in Table 1). During their follow-up visits, it was noted that the increased echogenicity in Patient 2 and splenomegaly in Patient 3 had resolved. On the other hand, although not present during diagnosis, Patient 4 was observed to have increased echogenicity in addition to hepatomegaly.

In GSD-IX patients, in initial visit a normal liver appearance was observed in Patient 15, while all other patients were detected to have an enlarged liver on liver ultrasound. Furthermore, increased echogenicity was observed in four patients (Patient 10, Patient 11, Patient 13, Patient 16) who had hepatomegaly (as shown in Table 1). During their follow-up visits, it was observed that three patients (Patient 8, Patient 10, Patient 13) had returned to normal ultrasound findings, while increased echogenicity had been added to the hepatomegaly findings in three patients (Patient 7, Patient 9, Patient 12).

#### Biochemical enzymes

The diagnostic basal values in five out of six patients with GSD-VI (83%), alanine transaminase (ALT) levels were elevated, while one patient (17%) had a normal ALT value. In the most recent visits, the ALT levels of all patients have returned to normal or near-normal levels. Additionally, it is worth noting that the ALT levels of Patient 4, which were high at diagnosis, have approached normal levels. On the other hand, all patients with GSD-IX had elevated ALT values, and Patient 15’s had an ALT level that was higher than the others. In four out of six patients with GSD-VI (67%), Aspartate transaminase (AST) was elevated at diagnosis, while two patients (33%) had normal AST values. In the most recent visits, a decrease in AST values was observed in all patients. In GSD-IX patients, initial AST was elevated in eight out of ten patients (80%), while one GSD-IXb patient and one GSD-IXc patient (20%) had normal AST values (Table 1).

Normal biotinidase enzyme activity has been observed in all patients diagnosed with GSD-VI and GSD-IX: both at the diagnosis and during their last visits.

Hypertriglyceridemia was present in all patients with GSD-VI, except for Patient 3. In patients with GSD-IX, hypertriglyceridemia was present in all patients, except for Patient 10, 11, 14, and 15 who had GSD-IXb. During the last visit, triglyceride levels in patients with GSD-VI had returned to normal or near-normal levels. A similar pattern was observed in patients with GSD-IX, except for Patient 13, who had an increase in triglyceride levels.

#### Liver biopsy

Out of six patients with GSD-VI, biopsies were performed on four patients (66%) while two patients (33%) did not receive biopsies. Among the patients who underwent biopsies, suggestive GSD was seen in all patients, but fibrosis was also observed in two patients (Patient 2 (14 years) and Patient 4 (12 years)). Out of 10 patients with GSD-IX, biopsies were performed on seven patients (63%), while three patients (27%) did not receive biopsies. Among the patients who underwent biopsies, suggestive GSD was seen in all patients, but fibrosis was also observed in two patients (Patient 12 (9 years) and Patient 10 (19 years)) as shown in Table 1.

#### Molecular genetic investigations(analysis)

In this cohort study, molecular genetic investigations were conducted on patients with GSD-VI and GSD-IX, confirming the diagnosis. In the GSD-VI patient group, three variants in the *PYGL* gene were detected. These include c.1131C>G (p.Asn377Lys), which was previously identified, and two newly discovered variants: c.1164G>A (p.Trp388*) and c.1806G>C (p.Arg602Ser). Specifically, new variants in the *PHKA2* gene, c.1324G>A (p.Val442Ile) and c.321C>A (p.Trp1067*), have been identified. Similarly, in the *PHKB* gene, new variants c.594+4T>G, c.1564C>T, and c.305+1G>A have been detected. Additionally, a new variant in the *PHKG2* gene, c.112G>A (p.Val38Ile), has been found. The variant *PHKB* gene c.1969C>T(p.Gln657*) has also been detected in three patients, but it was previously known (Table 1).

#### Echocardiography and electrocardiogram

In three patients with GSD-VI, echocardiography and electrocardiography were performed, and the results were found to be normal. In patients with GSD-IX, all patients underwent echocardiography and electrocardiography, and one patient was diagnosed with Patent Foramen Ovale (PFO) (Patient9) and another patient was diagnosed with Mitral Valve Insufficiency (MVI) (Patient13). The echocardiogram results of the other patients were normal, and all patients had normal electrocardiogram results.

#### Nutritional assessment

**Fig. 3:**
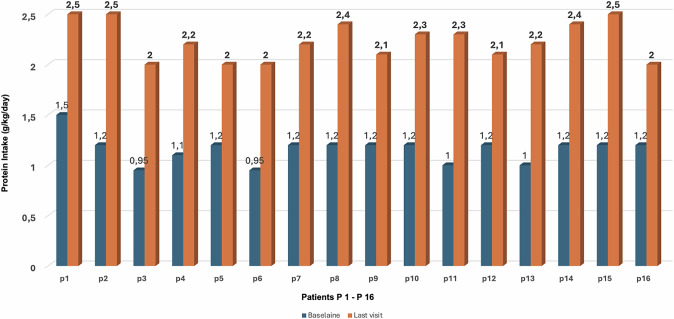
Protein intake (g/kg/day) per patient at baseline and final visit (patients P1-6 diagnosed with GSD-VI and P7-16 with GSD-IX).

In the nutritional assessment of GSD-VI patients, protein intake at the first visit was found to be 1.15 g/kg/day ±0.20, while in the dietary records from the last visit, it was determined to be 2.2 g/kg/day ±0.24 (Fig. 3). This reflects an increase of 1.05 g/kg/day, corresponding to a 91.3% increase in protein intake over the course of the study. At the last visit, more than 50% of the protein intake for patients with GSD VI was derived from animal sources. Protein intake for patients with GSD VI was distributed throughout the day, with protein consumed at each meal and snack, before bedtime, and prior to physical activities.

The uncooked cornstarch (UCS) treatment was initiated at 1 g/kg/day and subsequently reduced to 0.71 g/kg/day, resulting in a 29% decrease in dosage (Table 1). UCS was initiated at a higher dose, and once blood glucose levels were stabilized, the dosage was gradually lowered according to tolerance. Moderate amounts of dairy and fruits were permitted in the diet for patients with GSD VI. However, foods high in simple sugars were consumed in limited quantities.

For patients with GSD VI, caloric intake from fats was initially around 34.1% at the baseline and decreased to 30% by the last visit. Saturated fats contributed to less than 10% of the total caloric intake, and cholesterol intake was restricted to less than 300 mg per day. The medium-chain triglycerides (MCT) oil was used in 83% of patients with GSD VI (5 out of 6 patients).

Protein intake in patients with GSD IX, at the first visit was measured at 1.16 ± 0.08 g/kg/day, and by the last visit, it had risen to 2.25 ± 0.16 g/kg/day (Fig. 3). This reflects an increase of 1.09 g/kg/day, equating to a 94% rise in protein intake throughout the study period. By the time of the final assessment, animal-derived proteins accounted for at least 50% of the total protein intake. In this protein intake was also spread throughout the day, ensuring that protein was consumed at every meal and snack, before bedtime, and prior to engaging in physical activities.

The average UCS intake in patients with GSD IX initially started at 0.85 g/kg/day (Table 1). However, by the time of their last visits, the average intake had decreased to 0.52 g/kg/day, representing a reduction of approximately 60% over the course of the study. Treatment began with an elevated UCS dose, which was reduced after stabilization based on blood glucose levels and the patient’s ability to tolerate the adjustment. Notably, UCS was completely discontinued in four patients (P7-P9-P14-P15), while one patient (P16) never used it at all. In the diet, moderate consumption of dairy and fruits was allowed. Nonetheless, it was recommended to limit the intake of foods rich in simple sugars.

Fat intake in patients with GSD IX, as a percentage of total calories was reduced from 35.3% at the start to 32.5% by the final visit. Saturated fats made up less than 10% of the overall caloric intake, and daily cholesterol consumption was kept below 300 mg. MCT oil was administered to 20% of patients with GSD IX (2 out of 10 patients), reflecting its more limited use in this group (Table 1).

We implemented nutritional therapy with a high protein intake of 2–3 g protein/kg/day for an average duration of 4.5 ± 1.77 years.

## Discussion

We have reported on a cohort of 16 patients from a single center who have been diagnosed with either GSD-VI or GSD-IX and follow-up for about 8 years. Of these patients, 37% have been diagnosed with GSD-VI while the rest have been diagnosed with GSD-IX. The Turkish Statistical Institute has reported that the general rate of consanguineous marriage in Turkey over the last ten years is between 5.9% and 3.9%, with a maximum of 18.4% [10]. In our cohort study, we have observed that 62.5% of individuals with GSD-VI and GSD-IX diseases are the children of consanguineous marriages. As is the case with many pediatric diseases, consanguineous marriage poses a significant risk for GSD diseases.

In previous studies on GSD-VI, it has been observed that all [9] or a significant majority of patients present [1, 4, 5, 11] with hepatomegaly as a precursor symptom. In our cohort study, all GSD-VI patients also presented with hepatomegaly as a precursor symptom. As for GSD-IX patients in our cohort, we observed hepatomegaly in 90% of cases.

Additionally, short stature and hypoglycemia are expected to be observed in these patients. When the standard deviation (SD) for short stature is <−2/below 3 percentile in relation to the population, it is defined as a condition. This can be an initial symptom in patients with GSD-VI and GSD-IX. In a cohort study that included patients with GSD-III, GSD-VI, and GSD-IX, short stature was observed in 29% of the patient [9]. Another study demonstrated that 18% of patients with GSD-VI had this condition, but with dietary treatment, it has decreased to 7% [12]. Our own cohort study found that half of our patients with GSD-VI and GSD-IX patients had short stature. The proportion of patients with short stature who overcame the condition through nutrition was 67% for GSD-VI and 60% for GSD-IX.

According to the study findings, hypoglycemia was reported in <30% of GSD-IXa patients [9, 13–21], <35% of GSD-IXb patients [13, 16, 18, 20, 22, 23], <50% of GSD-IXc patients [13, 14, 16, 20, 24–26], and <40% of GSD-VI patients [4, 5, 7, 8, 11, 16]. In our cohort study, hypoglycemia presentation was observed in less than 20% of cases. In symptomatic cases of hypoglycemia, patients may experience various symptoms such as palpitations and sweating [27]. However, in patients GSD type VI and IX, ketotic hypoglycemia may occur, which can be asymptomatic and may be overlooked in daily life. It is well established that ketotic normoglycemia [28] was considered as a marker of hypoglycemia patients with GSDs. Since we adjust the diet by lowering the UCS and increasing protein intake, it is essential to carefully monitor for both ketotic normoglycemia and hypoglycemia.

Cardiomyopathy is extremely rare for GSD-VI and GSD-IX. Only two cases with cardiomyopathy accompanying GSD-VI and GSD-IXb have been described in the literature [9]. In our cohort study, no heart disease was detected except for Patent Foramen Ovalle (PFO) and Mitral Valve Insufficiency (MVI), which are not directly related to GSDs. Further research is needed to understand the relationship between GSD-VI, GSD-IX, and the heart. In chronic liver diseases, fibrosis is much more common than cirrhosis. In our cohort study, we did not observe any patients with cirrhosis. Among patients with GSD-VI, liver biopsy was performed in 4 of 6 patients (67%), with fibrosis detected in 2 (50% of those biopsied). For patients with GSD-IX, biopsy was conducted in 7 of 10 patients (70%), with fibrosis identified in 2 (29% of those biopsied). Another cohort study found that fibrosis was present in 48% of patients with GSD-IV and GSD-IX, while the prevalence of cirrhosis was less than 5% [9]. Although the exact relationship between age and the development of fibrosis is not fully understood, age is believed to be correlated with the development of fibrosis in chronic liver diseases [29].

Studies have shown a correlation between genotype and phenotype in fibrosis and cirrhosis [30]. It has been observed that liver fibrosis/cirrhosis is highly prevalent in patients with GSD IXc (caused by mutations in the *PHKG2* gene), with a rate of 95.8% [31]. Due to the severity of the phenotype in GSD-IXc, cases of liver transplantation have been reported, whereas this is not the case for other types of GSD-IX or GSD-VI [24]. Consistent with our cohort study, in the research presented by Roscher et al., only one patient exhibited fibrosis among those diagnosed with GSD-Ixc [9]. On the other hand, GSD-IXa (caused by mutations in the *PHKA2* gene) is a more prevalent type of GSD-IX that typically presents with milder symptoms and is generally not accompanied by fibrosis. However, Tsilianidis et al. reported two cases where fibrosis was present in GSD-IXa patients [20]. In our cohort study, we did not observe fibrosis in patients with GSD-IXa, while Roscher et al. documented cases of fibrosis in their study of GSD-IXa patients [9]. Although GSD-IXa is not as severe as GSD-IXc, it is crucial to consider the potential presence of fibrosis in GSD-IXa patients. Patients with GSD-IXb(caused by mutations in the *PHKB* gene) also have a mild disease course similar to that of GSD-IXa patients. It has been reported that 98% of patients with GSD-IXb experience hepatomegaly [32], which is consistent with our cohort study wherein all the GSD-IXb patients presented with hepatomegaly. However, Beyzaei et al. reported a case with a dual mutation in GSD-IXb that resulted in a severe disease course [32]. In a study by Zhan et al. reporting 42 patients with GSD-VI caused by mutations in the *PYGL* gene, it was observed that 96.7% of the patients had hepatomegaly [33]. In addition, in our own cohort study, all GSD-VI patients had hepatomegaly while fibrosis was found to accompany the disease in 33% of patients. Similar rates were also reported by Aeppli et al. in their study [1].

In the era of precision medicine, personalized nutrition therapy is crucial for the prognosis of a disease. As in our study, adherence to the prescribed diet directly affects the prognosis of the disease. The primary goal is to prevent conditions such as hypoglycemia and ketosis that can occur in GSD-VI and GSD-IX patients. Although there is no consensus on the exact composition of the diet, Kishnani et al. have published a suggestive guide for the nutrition of GSD-VI and GSD-IX patients [3]. In this study, it is recommended to start giving UCS to children over 6 months old at a small dose and gradually increase it, while our cohort study shows a high proportion of patients whose dose was either later reduced or not changed at all in our daily practice we prefer to start with a little bit higher dose, and then increasing the family knowledge for disease and nutrition, gradually reduce dose aiming the stay on minimal dose providing normoglycemia. Additionally, Koch et al.‘s review study on GSD-VI management highlights the importance of UCS use but does not provide a clear opinion on the dosage [34]. Considering these situations, we recommend individualizing the use of UCS for each patient to avoid the risks of overdosing or receiving an insufficient dosage. A high protein diet is important in conjunction with UCS use, and Kishnani et al. have provided three different approaches to explain why it is important: amino acids from protein can be used to make glucose, protein can directly fuel muscles, supporting their growth and repair, and replacing some carbohydrates with protein can reduce glycogen storage [3]. Considering the favorable prognosis of these diseases, these approaches are supported.

According to Kishnani et al., a diet should be high in protein and provide 2–3 grams of protein per kilogram of body weight or roughly 20–25% of their total daily caloric intake. Carbohydrates should provide approximately 45–50% of total calories, while fats should provide around 30% of total calorie [3]. When we examined the nutritional status in our cohort study, it was consistent with this suggestion and the patients showed a good prognosis. It should also be noted that an increase in carbohydrate consumption can lead to the development of carbohydrate-induced hypertriglyceridemia, particularly when consuming high-carbohydrate foods that contain simple sugars. To prevent this condition, it is preferable to consume complex carbohydrates. Although our cohort study included patients with hypertriglyceridemia, we cannot directly attribute their condition to their diet. However, this condition should be taken into consideration when providing nutrition therapy.

The use of MCT oil and anaplerotic agents in managing inborn errors of energy metabolism is gaining recognition, especially in the context of GSDs VI and IX. In line with this, MCT oil was utilized in 5 out of 6 patients with GSD VI, while it was administered to 2 out of 10 patients with GSD IX.

This study has several limitations: data have retrospectively been retrieved from paper files and blood β-hydroxybutyrate concentrations measured by a portable blood ketone meter were not used.

## Conclusion

Our cohort study of 16 patients with GSD-VI and GSD-IX provides valuable insights into the clinical presentations, molecular characteristics, and treatment outcomes of these diseases. The high prevalence of consanguineous marriages in our cohort highlights the importance of genetic counseling in populations with high consanguinity rates. We identified eight new variants, further expanding the spectrum of genetic variations associated with these diseases. Hepatomegaly, short stature, and hypoglycemia were common clinical features, with nutrition therapy playing a crucial role in improving patient outcomes. We emphasize the importance of individualized nutrition treatment plans, increasing the daily protein intake and trying to keep the UCS at the minimum effective dose.

## Data Availability

Additional data are available from the corresponding author on reasonable request.
